# *Impak Sihat*: A telehealth system development and feasibility evaluation to empower rural population in Malaysia on the quality use of medicines

**DOI:** 10.1371/journal.pdig.0000937

**Published:** 2025-08-07

**Authors:** Nor Ilyani Mohamed Nazar, Norny Syafinaz Ab Rahman, Nor Elina Alias, Syahrir Zaini, Tg Karmila Tg Mohd Kamil, Nurjasmine Aida Jamani, Mohamed Hassan Elnaem

**Affiliations:** 1 Department of Pharmacy Practice, International Islamic University Malaysia, Kuantan, Malaysia; 2 Department of Family Medicine, Kulliyyah of Medicine, International Islamic University Malaysia, Kuantan, Malaysia; 3 School of Pharmacy and Pharmaceutical Sciences, Ulster University, Coleraine, United Kingdom; Iran University of Medical Sciences, ISLAMIC REPUBLIC OF IRAN

## Abstract

The escalating global burden of chronic diseases necessitates innovative approaches to enhance medication adherence and quality use of medicines (QUM), particularly in underserved rural populations. This study developed and evaluated *Impak Sihat*, a telehealth system tailored to address systemic healthcare barriers in rural Malaysia through a three-phase mixed-methods design. Phase 1 involved qualitative interviews with 15 villagers, revealing smartphone ownership, inconsistent internet connectivity, high social media engagement, and limited critical appraisal of online health information. Phase 2 utilised these insights to create a dual-component system: a public portal with Malay-language educational materials, appointment booking, and a practitioner platform featuring secured patient data management. Phase 3 assessed feasibility via community demonstrations with 77 participants (mean age 53.4 ± 11.8 years), showing high acceptance scores (73–87%) across six domains. Key findings included strong usability (87.0 ± 16.3) and interface design (74.8 ± 23.9), though older adults scored significantly lower on interface design for learnability (ρ=−0.29, *p* < 0.01). The system’s offline functionality and WhatsApp integration mitigated rural connectivity constraints, yet challenges persisted in data confidentiality (lowest score: 73.1 ± 26.7). Healthy participants consistently rated the system significantly higher across multiple domains (Interface Design: p = 0.003, User Experience: p = 0.018, Healthcare Delivery: p = 0.002, and Overall Satisfaction: p = 0.003). These results underscore the potential of context-specific telehealth systems to bridge urban-rural health disparities while highlighting critical implementation barriers. This work highlights the importance of engaging key stakeholders, such as healthcare providers and community leaders, to ensure system sustainability and scalability. Overall, the study demonstrates that digital health interventions, when appropriately tailored to the specific needs of rural populations, can significantly contribute to reducing healthcare disparities and promoting patient empowerment.

## Author summary

In rural Malaysia, many face challenges in managing chronic diseases due to limited healthcare access, unreliable internet, and gaps in health knowledge. To address this, we developed *Impak Sihat*, a telehealth system designed with and for rural communities. By first listening to residents, we learned about their smartphone use, social media habits, and struggles with health information. We developed a system featuring offline educational materials in the local language, WhatsApp-based appointment booking, and secure tools for healthcare providers. Testing with 77 participants showed strong acceptance, particularly among younger and healthier users, though older adults found the interface less intuitive. While offline features helped bridge connectivity gaps, internet reliability and data security concerns persisted. Our work highlights how tailored digital solutions can empower rural populations by improving access to care and reducing travel burdens. However, success requires collaboration with local leaders, simplified designs for older users, and infrastructure investments. This study offers a model for using technology to address healthcare disparities, emphasising the importance of community-centred innovation in low-resource settings.

## 1. Introduction

The global increase in chronic diseases underscores the critical need for quality use of medicines (QUM) and robust medication adherence to ensure effective disease management. QUM emphasises the rational selection, safe use, and continuous monitoring of medications to minimise misuse, overuse, and underuse while empowering patients to manage treatment complexities, such as side effects and polypharmacy [1]. Effective QUM management by pharmacists and other healthcare practitioners could positively affect patients’ clinical outcomes and improve the healthcare system’s organisation [2]. In Malaysia, medication nonadherence rates remain alarmingly high, particularly in rural regions, where 50–80% of chronic disease patients report inconsistent adherence to prescribed therapies [3]. This disparity is exacerbated by systemic challenges, including limited access to healthcare facilities, socioeconomic constraints, lower health literacy, and infrastructural deficiencies such as unreliable internet connectivity [4,5] Rural populations often face compounded barriers, such as long travel distances to clinics, financial burdens from lost wages during medical visits, and a reliance on informal health information sources—factors that collectively undermine QUM and perpetuate poor health outcomes [6,7].

Medication adherence challenges in rural Malaysia manifest through four interconnected dimensions: socioeconomic constraints, technological limitations, cultural and behavioural factors, and systemic healthcare shortcomings [8,9]. While cost affects nonadherence rates, more pervasive barriers include insufficient medication understanding and a lack of essential drug information from providers, which were more pronounced among rural populations [10,11]. The travel burden for seeking healthcare service is a significant challenge for rural residents compared to those in urban areas, compounding transportation costs and time burdens [12]. Engaging and empowering rural communities through health education and telehealth services is essential. These services can significantly reduce medication errors and adverse drug events while fostering long-term sustainability.

While telehealth has emerged as a transformative tool to bridge healthcare gaps in remote areas, its implementation in Malaysia’s rural communities remains suboptimal. In Malaysia, the telemedicine blueprint was evidence of the government’s commitment to harnessing the potential of information and communication technology (ICT) to promote more equitable access to healthcare services for rural and remote areas [5]. Digital Health Malaysia (DHM), previously known as the Telemedicine Development Group (TDG), was formed in 2015 following the first telemedicine conference [13]. It is a platform where healthcare professionals, researchers, and the industry collaborate to advance the digital health agenda. However, despite its perceived advantages, digital health initiatives and services have not successfully reached and benefited the rural population of this country [5]. Despite governmental initiatives like the Telemedicine Blueprint and Digital Health Malaysia, existing telehealth systems have struggled to address the unique needs of rural populations, such as low digital literacy, cultural preferences for in-person care, and infrastructural limitations [5,14]. Prior studies highlight a paradox: while telehealth is recognised for its potential to improve medication adherence and health outcomes, rural healthcare providers and patients in Malaysia exhibit low readiness and trust in these platforms [15].

Furthermore, most telehealth interventions in low-resource settings focus narrowly on consultation services, neglecting integrated features like patient education, appointment management, and culturally tailored content—elements critical for fostering QUM and long-term adherence [16,17]. Engaging and empowering rural communities through health education and telehealth services is essential. These services can significantly reduce medication errors and adverse drug events while fostering long-term sustainability. However, current telehealth systems often fail to address the specific needs of rural populations.

The research gap lies in the mismatch between existing telehealth solutions and rural Malaysia’s technological landscape. Urban-centric platforms fail to accommodate unreliable internet coverage and low digital literacy among ageing populations. Current systems also neglect cultural preferences for WhatsApp-based communication and traditional medicine practices. No prior interventions have simultaneously addressed connectivity limitations through low-bandwidth design while integrating local language support and offline functionality. This study aims to bridge these gaps through three objectives: 1) Identifying rural-specific adherence barriers through qualitative analysis of internet access patterns and health information-seeking behaviours, 2) Developing *Impak Sihat* - a tailored telehealth system featuring mobile-optimised interfaces, downloadable Malay-language educational materials, and WhatsApp-integrated consultation tools, and 3) Evaluating system feasibility through community demonstrations measuring usability and socioeconomic inclusivity.

## 2. Methodology

The study has been registered and approved by the IIUM Research Ethics Committee (IREC 2024–194). Participation in all phases was entirely voluntary, and written informed consent was sought from all willing to participate. The study was divided into three (3) main phases, and different study designs were applied. The study phases are detailed below: -

### 2.1. Qualitative pre-development phase exploring internet access and health information-seeking behaviours

Fifteen participants from the local community were recruited via a convenient sampling method and interviewed using an open-ended survey to explore the community’s internet accessibility or issues and the use of the internet or social media for searching for health-related information. Participants were purposively selected from among community leaders and committee members, as they are considered knowledgeable representatives capable of providing informed responses on behalf of the broader village population. The findings have reached saturation with 15 participants. The responses were coded and analysed via thematic analysis. Significant emerged themes were considered and integrated into the development of the *Impak Sihat* telehealth system. This phase timeline spans from April 2024 to May 2024.

### 2.2. Development of the telehealth system (Impak Sihat) based on identified needs

**Fig 1:**
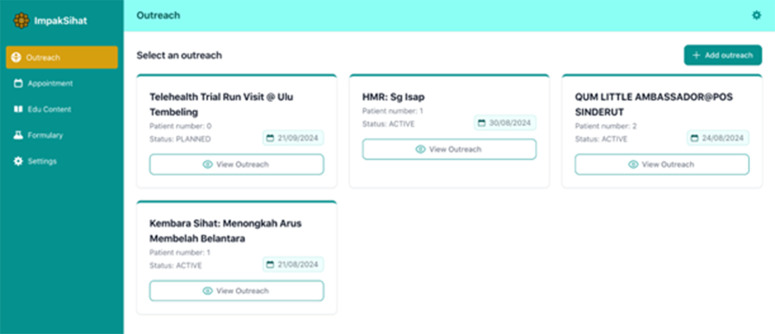
Outreach data management and monitoring.

**Fig 2:**
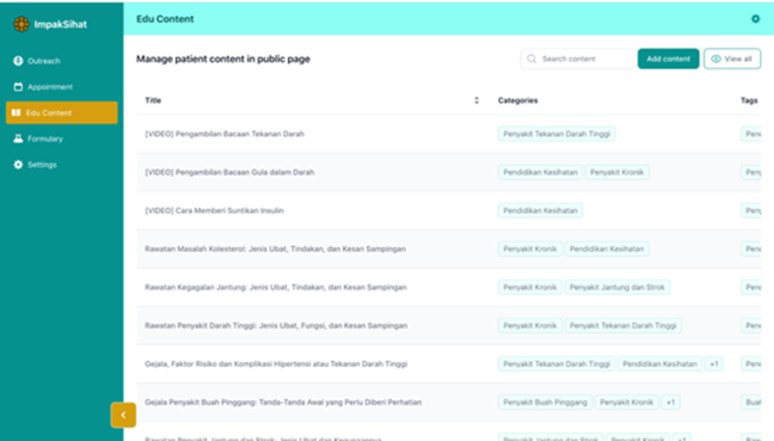
Educational materials management and update.

**Fig 3:**
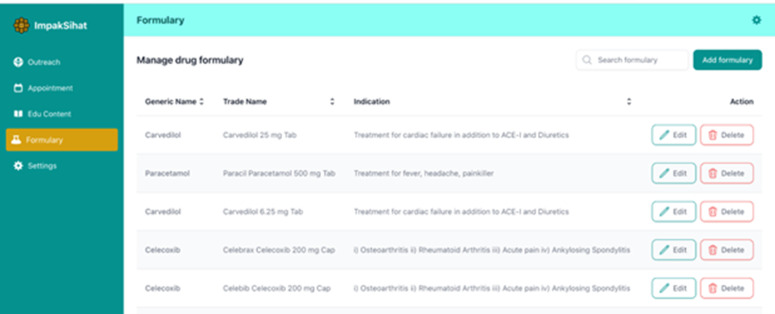
Medication Formulary available in Malaysia.

The telehealth system was developed by a local system development vendor with a healthcare background, experience, and familiarity with the healthcare system data. A research assistant was appointed to create educational materials (videos/posters/infographics) to be incorporated into the system. The team members/experts carefully reviewed the materials to ensure their usability among rural communities. The timeline for this phase spans from June 2024 to September 2024. The system was tailor-made to suit the needs of the rural population in Malaysia with simplified, lay language and user-friendly features. A series of meetings and a system demonstration session by the vendor to the research team were regularly conducted to ensure that the system was congruent with the expected needs of the team members and the rural population. The system has two main components: i) Health Education Portal & Appointment Booking System for public users and ii) Remote Health Monitoring and Patient Data Management System for administrators. The system incorporated special features on patients’ health education and appointment booking to ease the communication between patients and healthcare practitioners. We include illustrations of the system’s key features of outreach data management and monitoring (Fig 1), educational materials management and update (Fig 2), and medication formulation available in Malaysia (Fig 3).

*Impak Sihat* employs multi-layered security protocols in compliance with Malaysia’s Personal Data Protection Act (PDPA) to ensure effective data protection. Role-based access controls (RBAC) limit practitioner permissions. Two-factor authentication (2FA) via SMS/email is required. Patient data for analytics is pseudonymised, and audit trails log all activities. Biannual third-party penetration testing identifies and addresses vulnerabilities [18]. The simplification of health data was executed through a structured workflow in which seven pharmacists and two physicians translated clinical guidelines into clear, accessible language (for example, replacing “antihypertensive” with “blood pressure medicine”). The content featured visual aids (such as dosage icons) and culturally relevant examples (like dietary recommendations based on local foods). The simplified materials underwent validation by rural users, with revisions made based on their feedback regarding clarity [19].

### 2.3. Post-development feasibility evaluation of the Impak Sihat system through community demonstration and validated questionnaires

Once the system was about 80% complete, a feasibility study was conducted via telehealth system demonstration directly to the potential users among the rural population. A questionnaire was adapted and translated into Malay from the Telehealth Usability Questionnaire (TUQ) [20]. Five experts among the healthcare practitioners and scholars in the field were invited to review the content validity of the translated version. The content validity index (CVI) rated by the five experts was 0.919, and the Cronbach alpha value was 0.917, which shows good validity and internal consistency of the questionnaire. The validated questionnaire was distributed to the participants post-demonstration for feedback on usefulness, reliability, learnability, user experience, and future use recommendations. The inclusion criteria for the participants were being more than 18 years old, signing the informed consent form, being able to read and write, and owning or using at least one smartphone or any other communication device per household. The sample size was 78 respondents, calculated and estimated using Raosoft software (http://www.raosoft.com/samplesize.html) with a population size of 400 people, 95% confidence interval, 10% margin of error and 50% response distribution. The timeline for this phase spans from September 2024 to November 2024.

During the demonstration session, the participants were systematically introduced to the system’s essential features, mainly health education, appointment booking, and live video calls with healthcare practitioners. They were taught how to search for health information using the keywords on their mobile devices, set their appointments, and experience actual video calls with healthcare practitioners.

### 2.4. Statistical analysis

Statistical analyses were performed using SPSS version 29. Python (version 3.11) was used to create illustrative visualisations. Descriptive statistics were calculated for all domain scores presented as means and standard deviations. The normality of score distributions was assessed using Shapiro-Wilk tests. Consequently, non-parametric tests were employed: Spearman’s rank correlation coefficient (ρ) was used to examine relationships between domain scores and continuous variables (e.g., age), while Mann-Whitney U tests were used for categorical comparisons (e.g., gender, health status). Correlations between the six evaluation domains (System Usability, Interface Design, Technical Performance, User Experience, Healthcare Delivery, and Overall Satisfaction) were analysed using Spearman’s correlation coefficients. The strength of correlations was interpreted as weak (ρ < 0.3), moderate (0.3 ≤ ρ < 0.6), or strong (ρ ≥ 0.6). All statistical tests were two-tailed, and p-values less than 0.05 were considered statistically significant.

## 3. Results

### 3.1. Key themes identified from the pre-development phase activity

#### 3.1.1. Availability of smartphone devices with inconsistent internet coverage.

Most respondents owned at least a smartphone (Android) in a household unit and used their data (4G) for internet browsing, which cost them between RM30 and 45 per month. However, the internet speed test and coverage could have been more consistent in different areas of the villages and with varying weather conditions. This finding provides insight into the later implementation of telehealth services in this rural area, whereby external internet services might be beneficial to be introduced where it provides internet connectivity through a network of satellites, allowing users to access the internet in areas where traditional wired or cellular internet access may be limited or unavailable. Other than that, the identified high-speed internet areas of the village should be further identified and gazetted as telehealth terminals for the local community, especially for live-streaming consultation.

#### 3.1.2. *Active use of social media.*

Most respondents were active on social media, and many had more than one (1) account, mainly WhatsApp, Facebook, Instagram and TikTok apps. They spend 1–10 hours browsing social media daily, primarily for leisure purposes - music, recipes, news, politics, socialising, and online shopping. Only a few local communities use the internet for work-related matters since the community’s economic activity is related to small-scale agricultural activities. Those who spend over 5 hours daily admit they love watching short videos, especially from TikTok. This information provides the team with meaningful insight into further engaging with the local community via social media platforms that must be linked with our telehealth system.

#### 3.1.3. *Health literacy and internet usage for health-related information.*

Health Literacy is the ability to access, understand, appraise, and use information to make healthier choices [21]. Six respondents from the local community admitted using the internet to find health information, such as symptoms of diseases, traditional medicine, massages and health tips. They needed clarification when further asked about the validity and reliability of the gained information. Still, most of the time, they tend to buy or try out any products/ recommendations/ advice/ tips they encounter on the internet. This is an important finding on the health literacy of this rural population and an opportunity for community education and empowerment from the provider’s perspective.

### 3.2. Telehealth system development

The system was developed using a low-bandwidth, mobile-first architecture to accommodate rural connectivity constraints. It has three key features: offline-mode educational content (downloadable PDFs/videos in Malay), WhatsApp API integration enabling SMS-based appointment reminders, and RBAC for healthcare practitioners, with audit logs tracking all patient data interactions.

Content Development involves mainly the educational materials of 50 infographics and 15 animated videos (≤3 minutes) on QUM, validated by seven pharmacists for health literacy. Furthermore, the appointment system that sends SMS reminders in Malay, with offline fallback options for no-internet users

The developed telehealth system has two important components: i) for the public and ii) for healthcare practitioners with login credentials. Public users do not need to register login credentials and can use the system immediately. Public users can freely browse and go through the educational materials on various health-related topics, including medication usage, disease prevention, and lifestyle management, which have been developed and integrated into the system as an educational portal. The developed materials were simplified, mainly using laymen’s terms and with more infographic designs. Another vital feature for public users is the ‘appointment-booking feature’. Through this feature, public users can make appointments and communicate with healthcare practitioners to ask about any health-related information to the healthcare practitioners in charge without the need to be physically present at the healthcare facilities.

The second component for healthcare practitioners is registering and uniquely creating their login credentials; they can only access patients’ data on disease progression, vital signs and laboratory investigations, medication prescriptions, dosage, and adherence. Security system development for patient data management is crucial to telehealth system development. The vendor has employed industry-standard security measures to safeguard patient data effectively. This includes implementing encryption techniques, access controls, and regular security audits to mitigate potential risks and vulnerabilities. Encryption techniques such as Transport Layer Security (TLS) have also ensured secure communication [18].

**Fig 4:**
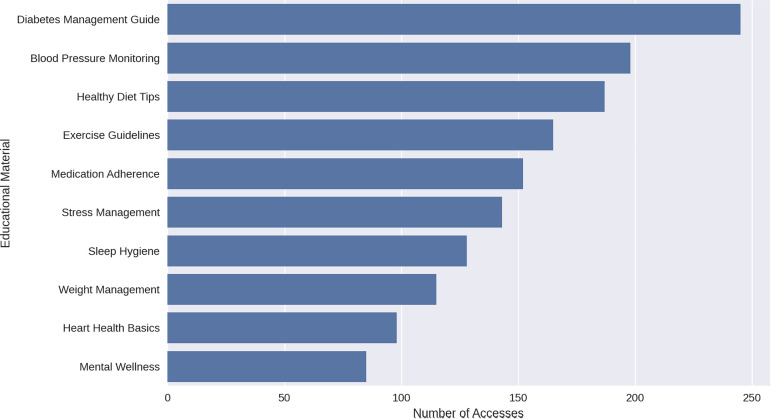
Most accessed materials.

**Fig 5:**
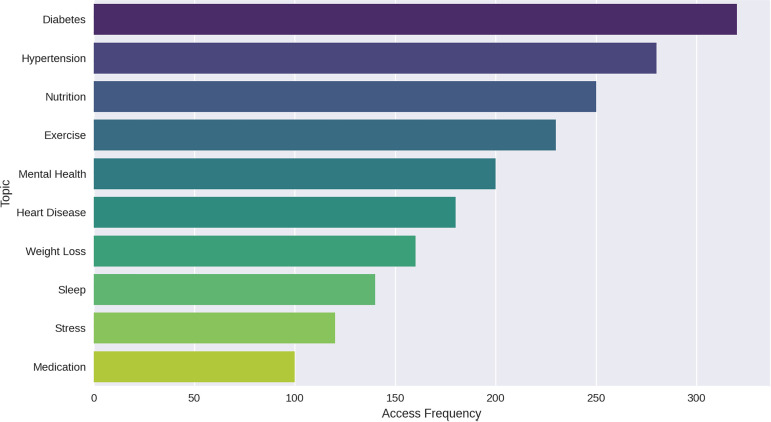
Popular topic distribution.

**Fig 6:**
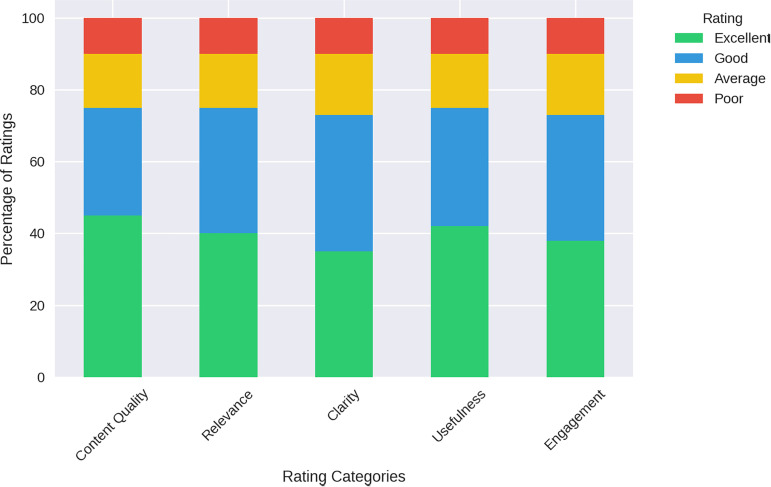
Content feedback and ratings.

**Fig 7:**
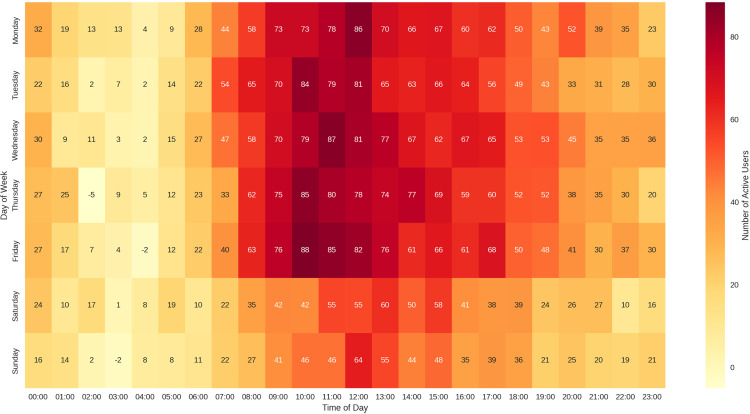
Weekly content consumption patterns.

The content performance analysis feature has been incorporated into the system. These features include:

i. Most Accessed Materials: This horizontal bar chart clearly shows the ranking of educational materials by access count, with Diabetes Management Guide being the most accessed content, followed by Blood Pressure Monitoring and Healthy Diet Tips (Fig 4).

ii. Popular Topics: This bar chart displays the frequency distribution of popular topics, showing Diabetes, Hypertension, and Nutrition as the most frequently accessed topics. This visualisation helps administrators identify trending subjects (Fig 5).

iii. Feedback and Ratings: This stacked bar chart shows the distribution of user ratings across different aspects of content (Content Quality, Relevance, Clarity, Usefulness, and Engagement). The colour-coded segments represent different rating levels (Excellent, Good, Average, Poor), allowing easy comparison across categories (Fig 6).

iv. Content Consumption Patterns: This heatmap visualises user activity patterns throughout the week, with darker colours indicating higher usage (Fig 7).

These visualisations provide administrators with clear, actionable insights into content popularity and access patterns, user preferences and interests, quality metrics across different aspects of content and temporal patterns of system usage.

### 3.3. Feasibility study on users’ acceptance and experience

In this feasibility study, the local community participated in a demonstration session of the telehealth system, and their feedback was collected via a validated questionnaire.

#### 3.3.1. *System demonstration implementation.*

A telehealth system demonstration and feasibility evaluation were conducted at two rural villages of Ulu Tembeling, Kg. Kuala Sat and Kg. Bantal in the District of Jerantut Pahang. Seventy-seven patients who met the inclusion criteria consented to the telehealth demonstration session. Fifty-one (66.2%) respondents were female, and another 33.8% were male. The mean age was 53.4 (±11.8) years old. Most respondents were housewives (48.1%) for females and self-employed or working in agricultural sectors for males (39.0%). Table 1 lists the summary of demographic data collected.

**Table 1:** Demographic data of the local respondents who participated in the post-development study.

Demographic variables	
Gender, n (%) -Male -Female	51(66.2) 26 (33.8)
Age, mean (+sd)	53.4 (±11.8) years old
Job category, n (%) -Housewife -Self-employed -Agricultural sector -Government sector -Business/industrial sector	37(48.1) 16(20.8) 14(18.2) 5(6.5) 4(5.2)
Monthly income, mean (+sd)	RM 441.04 (+451.4)
Education, n(%) -Primary -Secondary -Tertiary	37(48.1) 24(31.2) 2 (3.6)
Marital status n(%) -Married -Non-married -Divorced	59(76.6) 7 (9.1) 11(14.3)
Health status n (%) -With chronic disease -Without chronic disease	38(49.4) 39(50.6)
Treatment status n (%) -On long-term treatment -Without long-term medication/ treatment	35(45.5) 42(54.5)
Time spent for internet browsing per day n(%) - < 2 hrs -2–5 hrs -5–10 hrs - > 10 hrs	54(70.1) 15(19.5) 7(9.1) 1(1.3)
Monthly internet data subscription, mean (sd)	RM27.23 (+23.30)

#### 3.3.2. *Feasibility Evaluation.*

The feasibility evaluation was conducted across six key domains (Q1-Q6), with scores ranging from 0 to 100:

- Q1 (**System Usability**- The usefulness of the Telehealth System): Mean score of 87.01 (SD = 16.25)
- Q2 (**Interface Design**- User-friendly features and ease of learning and understanding): Mean score of 74.78 (SD = 23.99)
- Q3 (**Technical Performance** - Audiovisual quality of the system): Mean score of 75.32 (SD = 21.39)
- Q4 (**User Experience**- Interaction quality with healthcare practitioners.): Mean score of 80.71 (SD = 18.13)
- Q5 (**Healthcare Delivery**- Reliability of information retrieved from the system and data confidentiality): Mean score of 73.05 (SD = 26.66)
- Q6 (**Overall Satisfaction**): Mean score of 80.30 (SD = 22.54)

The relationships between the six domain scores (System Usability, Interface Design, Technical Performance, User Experience, Healthcare Delivery, and Overall Satisfaction) were examined using correlation analysis. First, the normality of score distributions was assessed using the Shapiro-Wilk test. As all domains showed significant deviation from normality (p < 0.001), Spearman’s rank correlation coefficient (ρ) was used. The strongest correlations were observed between:

1. Technical Performance and Healthcare Delivery (ρ = 0.679, p < 0.001)
2. User Experience and Healthcare Delivery (ρ = 0.618, p < 0.001)
3. Technical Performance and Interface Design (ρ = 0.607, p < 0.001)

The weakest, though still significant, correlation was between System Usability and Healthcare Delivery (ρ = 0.318, p = 0.005). This correlation analysis suggests strong internal consistency among the evaluation domains. All correlations were positive and statistically significant, indicating that improvements in one aspect of the system were generally associated with improvements in other aspects.

Based on the comprehensive analysis of associations between domain scores and demographics, the most notable findings were that health status emerged as the strongest demographic predictor, with healthy participants consistently rating the system significantly higher across multiple domains (Interface Design: p = 0.003, User Experience: p = 0.018, Healthcare Delivery: p = 0.002, and Overall Satisfaction: p = 0.003). Age showed a significant negative correlation only with Interface Design scores (r = -0.290, p = 0.010), suggesting older participants found the interface less satisfactory. Gender analysis revealed no statistically significant differences in domain scores between males and females, indicating the system’s gender-neutral usability. Education level analysis showed that participants with secondary education generally gave higher scores across most domains, though this trend was not statistically significant. These findings suggest that while the telehealth system demonstrates consistent performance across gender groups, special attention may be needed to optimise the interface for older users and those with existing health conditions.

## 4. Discussion

The findings of this study demonstrate that *Impak Sihat*, a telehealth system tailored to rural Malaysia’s socioeconomic and technological context, holds significant potential to improve medication adherence and empower chronic disease management through accessible health education and remote care. The system achieved high usability scores, underscoring its relevance in addressing unmet healthcare needs. However, challenges such as lower interface satisfaction among older adults and persistent connectivity barriers highlight critical gaps that reflect broader telehealth implementation struggles that could impact digital health equity in low-resource settings. By integrating offline functionality, local-language content, and robust security measures, *Impak Sihat* advances a model for balancing innovation with inclusivity—a necessary paradigm for sustainable telehealth adoption in underserved communities.

### 4.1. Community acceptance for rural telehealth initiatives

Health technology interventions are essential to rural healthcare services as they enhance residents’ access to care (including speciality care) that they would not otherwise be able to get in remote areas and reduce staffing costs, travel expenses, and travel time [17]. Rural residents either travel great distances to receive care, have limited access to healthcare, or are delayed in seeking care until after an emergency. Poor health outcomes and a financial and social burden on the patient and the healthcare system can arise from limited access to care. An additional strain is placed on the patient by the expense of travelling for medical care, including lost work hours, decreased productivity, and higher childcare or caregiver support [6]. Telehealth extends the reach of health services and provides the opportunity to reduce barriers to care in rural communities.

This study in a rural community in Malaysia revealed an overall positive acceptance and experience among the local community towards the newly developed telehealth system – *Impak Sihat*, which aims to provide health education and address the issues of chronic diseases and quality use of medicine among disease progression in the rural population. This is consistent with a similar study in a rural community, which found that 88% of respondents were either considering or favouring telehealth, and only 12% of respondents reported that they would not be open to telehealth [22]. Telehealth perceptions were generally favourable among rural patients and providers, although satisfaction was lower among older patients and providers. Another finding has strongly suggested that telehealth approaches may add value and efficiency to rural clinical practice [23].

### 4.2. Issues to consider before widespread adoption

Despite the positive and favourable acceptance of the current telehealth initiatives, several issues need to be addressed in our local rural setting before this initiative can be fully implemented.

#### 4.2.1. Aging population of the rural community.

The mean age of participants in this current study is 53 (±11.8) years old. The result also demonstrated a strong correlation between the age with user-friendly features and the ease of learning and understanding (p < 0.05), where the lower the age, the higher the index score for this domain. This is expected as many of those shown interest in using telehealth were from the younger generation [22]. A study in a rural population with 206 respondents (mean age was 60) found that video conferencing and patient portal use pose barriers to older people with less education. However, these barriers disappear when telehealth is available through the telephone. Being younger, married/partnered, and having some college education were significantly associated with patient portal use [24]. From the findings, it is significant to acknowledge the high prevalence of ageing in rural communities. Hence, the younger generation needs to be empowered by family members or the local community to assist the older people with telehealth services.

#### 4.2.2. *Socioeconomic status (SES).*

Low SES in rural Malaysia is a pronounced issue that presents significant challenges for individuals and communities. This study shows that most of the rural population has low SES, which might affect their ability to acquire additional cost of internet data if the telehealth service is provided to them. Hence, it should be designed as a web-based or with minimal data consumption if it is to be developed and implemented as a mobile application. Studies found that increasing age and being in the lowest median household income quartile were associated with lower odds of completing virtual visits overall [25,26]. From another perspective, the telehealth service has also benefitted the rural population by reducing travelling costs and improving access to healthcare facilities [7]. Hence, the government needs to play an important role in providing basic needs or facilities to the rural population, ensuring the sustainability of the service in the long run.

#### 4.2.3. Unstable internet access in the rural area.

One of the central themes from the qualitative findings of the current study is unstable internet access in an area that heavily depends on local weather. This remains a significant challenge that might hamper telehealth development and exacerbate healthcare disparities between urban and rural communities. Solving these challenges that affect system performance in rural regions requires a holistic and well-defined strategy. This study suggests the following crucial actions: the prioritised development and expansion of digital infrastructure by the government; the cultivation of strong collaborative activities between government and private sectors; a thorough investigation and deployment of satellite internet solutions, and the deployment of sustainable digital literacy training module to empower rural populations.

While urban regions in Malaysia enjoy high-speed internet connectivity of more than 90% coverage and advanced digital infrastructure, rural areas often struggle with inadequate internet services (<70% coverage) and poor facilities [27]. Rural areas are often located in remote regions that are difficult to reach, making it costly and logistically challenging for service providers to deploy the necessary infrastructure [28]. Additionally, the lower population density in these areas does not present a financially viable market for telecommunications companies, leading to a lack of competition and innovation [28].

Therefore, cutting-edge options like satellite internet and neighbourhood Wi-Fi projects can be explored. In remote locations where traditional infrastructure is impracticable, satellite technology may be able to reach people [29]. Community-driven initiatives can empower rural populations and encourage local ownership of digital resources by bringing local communities together to create Internet services [28]. Furthermore, digital literacy programs should be implemented to equip rural residents with the skills needed to navigate the online world effectively. By fostering digital skills, individuals can better utilise available resources, engage in e-commerce, and access online education and healthcare services [30,31].

#### 4.2.4. *Data reliability*, *confidentiality and trust issues.*

The mean score for the system’s information reliability and data confidentiality domain shows the lowest score among the six domains, with a 73.1% score. Data confidentiality and reliability have become critical principles in the digital age since information is readily available, and the internet is a major source of knowledge. Data reliability refers to the accuracy, consistency, and trustworthiness of information available in the system. The fast growth of social media platforms has significantly impacted how individuals consume and interpret information. Hence, it is essential to ensure that the available content of the telehealth system is from valid and reliable resources. This can be achieved by having a specific team develop and review the educational content regularly. Furthermore, the broader implementation of the system among community members may present risks related to the inaccurate reporting of clinical data, including blood pressure, pulse rate, blood cholesterol, and sugar levels, which may be measured by patients at home and eventually lead to misinterpretation of data by the healthcare practitioners [32]. Therefore, this study suggests that it is essential to identify and develop a core group of people within the community – community health workers who can undergo continuous training, enabling them to play a pivotal role in ensuring the reliability and effectiveness of the system at the local level [19].

While data reliability is a crucial component of the developed telehealth system, confidentiality is equally significant in shaping public perception. The internet has transformed the landscape of personal data collection, raising questions about how individuals’ information is stored, used, and protected. The public’s perception of internet data reliability and confidentiality has important societal ramifications [33]. A lack of trust in data confidentiality can lead to disengagement with the healthcare providers providing the telehealth service.

Conversely, heightened awareness of data confidentiality issues can empower individuals to take control of their digital footprints. As users become more informed about their rights and the importance of data protection, they may demand greater accountability from corporations and advocate for stronger privacy regulations. This shift in perception can foster a culture of data responsibility, where individuals and organisations prioritise ethical data practices.

#### 4.2.5. Integrating *Impak Sihat* into the mainstream healthcare in Malaysia.

The IMPAK SIHAT system has been developed to support rural communities and healthcare practitioners in Malaysia in managing chronic diseases. This objective aligns with the diverse capabilities of telehealth within the healthcare sector, including chronic disease management, continuity of care, medication adherence, and health education [34]. Telemedicine significantly improves patient outcomes, access, and satisfaction in chronic disease management, especially diabetes care. By overcoming geographical barriers and enhancing patient engagement, telehealth platforms have the potential to transform policies and public health strategies on global healthcare delivery [35]. Therefore, a coordinated effort involving key stakeholders—namely, the Ministry of Health Malaysia as the primary healthcare authority, academic institutions as system developers and educators, and the community as end-users—is essential to ensure the effective and impactful implementation of this telehealth system within rural populations. Although the successful delivery of telehealth services has been documented, widespread adoption remains limited due to regulatory, legal, and reimbursement challenges that must be addressed prior to full-scale implementation [36].

## 5. Conclusion & recommendation

The implementation of telehealth represents a potentially effective strategy to mitigate healthcare access disparities faced in rural Malaysia. The current analysis suggests an encouraging level of acceptance of the deployed telehealth system among rural community members. However, the system may require further modifications and enhancements before it can be implemented on a full scale. Numerous factors must also be evaluated and resolved before deployment to guarantee acceptance and efficacy across diverse age demographics and socioeconomic strata. Moreover, issues about inconsistent internet connectivity and information reliability and confidentiality warrant careful consideration. By investing in infrastructure, fostering innovative technological advancements, and enhancing digital literacy, Malaysia can strive to bridge the digital divide, ultimately facilitating more equitable access to healthcare services.
